# Protein malnutrition during lactation affects thoracic aortic tunica media thickness in Wistar rat pups

**DOI:** 10.1590/ACB361008

**Published:** 2021-11-29

**Authors:** Ronaldo Miguel Carvalho, Isabeliza Maria do Espírito Santo Rangel Ferreira, Fausto Miranda

**Affiliations:** 1 Fellow Master degree. Postgraduate Program in Interdisciplinary Surgical Science - Surgery Department – Universidade Federal de São Paulo (UNIFESP) – São Paulo (SP), Brazil.; 2 Fellow PhD degree. Immunology Department – Universidade Federal Fluminense (UFF) – Rio de Janeiro (RJ), Brazil.; 3 PhD, Full Professor, Chairman. Surgery Department – Universidade Federal de São Paulo (UNIFESP) – São Paulo (SP), Brazil.

**Keywords:** Protein Deficiency, Aorta, Thoracic, Malnutrition, Rats

## Abstract

**Purpose:**

To evaluate the morphological effects of a low-protein diet during maternal lactation on the offspring’s thoracic aorta.

**Methods:**

Two female Wistar rats were mated with male of the same species at 4 months of age. Until the birth of the pups, all animals received commercial rat chow. After giving birth, the puerperal females were divided into two groups and adjusted the litter to five puppies per group: a control group that received commercial feed, and an experimental group whose diet included the same amount of calories, but 8% lower protein content. All animals’ masses were measured throughout the lactation period, and the pups were euthanized after weaning at 21 days of age. The thoracic aorta was removed, histologically processed and stained with Weigert’s resorcin-fuchsin for histomorphometric analysis of tunica media thickness.

**Results:**

Although both groups were born with similar body mass, during the 21 days of lactation the restricted protein group gained only 39% of the body mass of the control group. Histomorphometric analysis revealed that the restricted protein group had a significantly lower mean tunica media thickness than the control group.

**Conclusions:**

A low-protein diet for nursing mothers influences mass gain and aortic tunica media thickness in their offspring.

## Introduction

Malnutrition is a public health concern particularly in economically disadvantaged regions. According to the 2017 Food and Agriculture Organization’s report, about 821 million people suffer from various forms of malnutrition in the world, being the children the most affected. Low-birth weight is correlated with perinatal, neonatal and postnatal morbidity and mortality, as well as chronic diseases in adulthood if the condition is not reversed^1^. An underweight mother in the pre-gestational and/or gestational period is a determining factor for low-birth weight. The persistence of this condition in puerperal women contributes to developmental delay in neonates^2^.

Since 1986, Barker and Osmond have studied the medical and biological consequences of perinatal nutrition, proposing that these consequences are closely linked to the origin and development of disease^3^^,^ 4.

The effects of overnutrition or malnutrition during the early stages of growth can alter cell physiology and development and could be associated with the onset of cardiometabolic diseases^5^. Thus, it is important to understand the body’s reactions and adaptations to different conditions and stimuli, such as malnutrition, especially since phenotypic changes, or epigenetics, can occur without genotype alterations.

Phenotypic plasticity, i.e., phenotype modification without DNA sequence alterations, occurs mainly through epigenetic change, such as DNA methylation, microRNA expression and histone acetylation^6^. Macro and micronutrients have been identified as important factors in epigenetic processes, such as DNA methylation, chromatin remodeling and amino acid exchange in the N-terminal portion of histones^7^^-^ 9. Altered concentrations of amino acids, such as methionine and cysteine, as well as the reduction of choline and folic acid in the diet can alter the DNA methylation process, leading to both hypermethylation and hypomethylation^10^.

A low-protein diet during pregnancy and lactation is associated with growth changes, asymmetric or undersized organs, increased systolic pressure, dyslipidemia and higher insulin concentrations in experimental animals^11^^,^ 12. Thus, it appears that the development of cardiovascular and metabolic diseases could be associated with a combination of two factors, such as exposure time to a low-protein diet and its characteristics during the postnatal period^6^^,^ 13.

A mother’s nutritional status influences the composition and concentration of macro and micronutrients in breast milk. Studies seeking to identify and evaluate the effects of such a diet on the breast milk of malnourished mothers are few, mostly assessing changes in malnourished mothers during pregnancy. The positive correlation between low weight in mothers and newborns and the influence of malnutrition on the structural composition of milk shows the importance of research on inadequate diet for human development. Rats are highly similar to the human genome, being used constantly as study models for hypertension, diabetes, obesity, cancer, age and nutrition^14^^,^ 15. Thus, our hypothesis was that maternal malnutrition during lactation causes structural changes in the aorta of offspring.

## Methods

The experimental procedures of this study were approved by the Ethics Committee Animal Use of the Universidade Federal de São Paulo (CEUA-UNIFESP) under protocol number 3159271117, and animal care and handling followed the Guide for the Care and Use of Laboratory Animals^16^ and Council for International Organizations of Medical Sciences (CIOMS) ethical code for animal experimentation^17^.

The Wistar rats, created and coming from the university *bioterium*, were housed in polypropylene cages (48 × 20 × 13 cm) in groups of two nulliparous females for one male at 25±2°C, under a 12/12-h light/dark cycle, with food and water *ad libitum*. They were mated at 120 days of age and received commercial feed (23% protein) until the birth of the pups. The division of the females and their pups was done after drawing with a sealed opaque envelope for the control group (C) or reduced protein (RP) group. The control group (C) had free access to water and normal diet (23% protein commercial feed), and the reduced protein group (RP) had free access to water and received diet with the same caloric content as the control group, but with lower protein content (8% protein).

The low-protein diet was prepared manually, and its composition is shown in Table 1. The protein source of this diet was macerated commercial ration, and the missing calories were compensated by adding cornstarch. The low-protein ration was supplemented with vitamins and minerals to obtain the same composition as commercial feed, based on U.S. National Research Council and National Institutes of Health recommendations^18^. The hypoprotein diet started to be provided to the RP group rat on the day of the pups’ birth, and the litters of both groups were adjusted to five puppies^19^.

**Table 1:** Composition of the normal and the low-protein diet * .

Nutrient		Diet	
	Normal protein	Kg/diet	Low protein
Total energy Kcal	4,070.4		4,070.4
Protein %	23		8
Carbohydrates %	66		81
Fats %	11		11
Protein g	230		80
Carbohydartes g	676		826
Fats g	50		50
Vitamin mixture g	4		4
Mineral mixture g	40		40

* The protein source (8%) of this diet was macerated commercial ration, and cornstarch was added to compensate the missing calories in the low-protein diet. Vitamin and mineral supplements were added to obtain the same composition as the commercial feed, which is based on U.S. National Research Council and National Institutes of Health recommendations^18^.

To assess the nutritional status of the animals, the amount of feed ingested and the body mass, both the pups and the matrices, were monitored daily throughout the experimental period. The ration was administered once a day, and the diet offered to the RP group had the same mass amount, measured in grams, as that offered to the group C. This value was obtained daily, by weighing the amount of ration eaten on the previous day by group C. The mass of each animal in each group was obtained daily with a precision scale of 1 gram until the end of lactation (day 21).

On day 21, the puppies of both groups were sacrificed with an intraperitoneal injection of 0.2 mg of sodium pentobarbital per 100 g of body weight. Then, the chest was opened, and the left ventricle was directly punctured for intracardiac perfusion with 0.9% saline solution and formalin. In addition, a direct puncture of the right atrium was performed for exsanguination. After the perfusion, the aortas were removed and fixed in 10% formaldehyde for 48 h, and, after fixation, the fragments were processed according to the histological processing routines. The segments were then embedded in paraffin. Histological sections were made in a microtome (Spencer 820^®^), with thickness of 10 μm from the middle third of the descending thoracic aorta, and were stained with Weigert’s resorcin-fuchsin dye.

Histomorphometric analysis was performed to quantify the aortic tunica media thickness of Wistar rat pups under different experimental conditions. The images were obtained using an Olympus BX41^®^ microscope and Analysis Get It Research Software^®^. Five aortic fragments from each animal were analyzed, and one image was taken (x200 magnification) of each section, totaling 25 images per group.

We used the software S-PLUS version 6.0^®^ for the statistical analysis of the results of the evolution of the body mass of the puppies during the experiment and evolution of the body mass of the matrices. These results were analyzed using the Mann-Whitney test. P <0.05 was considered significant. Mean, median, first and third quartiles, 95% confidence interval and standard deviation were analyzed.

Statistical software R-project version 3.51^®^ was used for the statistical analysis of the results of histomorphometry. The results are expressed as mean and standard deviation from the mean (SD) and were analyzed using the T-Student mean comparison test. P <0.05 was considered significant.

## Results

All animals were included in the evaluation of the results.

The descriptive analysis of body mass data in the control group of the puppies showed mean of 21.68, median of 22.06, SD of 12.12 and 95% confidence interval of 14.74–28.63. In the group with protein restriction of the pups, we obtained mean of 10.17, median of 9.81, SD of 3.67 and 95% confidence interval of 8.07–12.27. The comparison of groups with a 95% confidence interval resulted in p <0.01 (Mann-Whitney test).

The descriptive analysis of the body mass data of the matrix of the control group showed mean of 311.08, median of 301.50, SD of 24.59 and 95% confidence interval of 296.99–325.18. In the group with protein restriction, the matrix showed mean of 243.12, median of 244.50, SD of 22.88 and 95% confidence interval of 230.02–256.23. The comparison of groups with 95% confidence interval resulted in p <0.01 (Mann-Whitney test).

**Figure 1:**
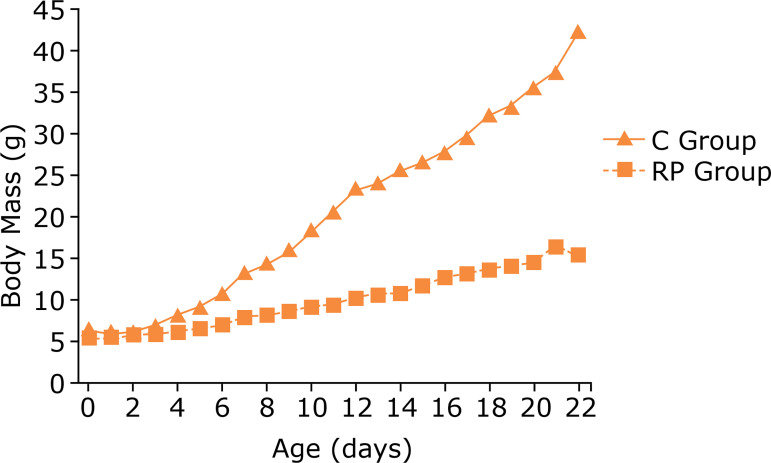
Evolution or pup body mass in the C and RP groups over the 21-day lactation period (p<0.01 Mann-Whitney Test).

**Figure 2:**
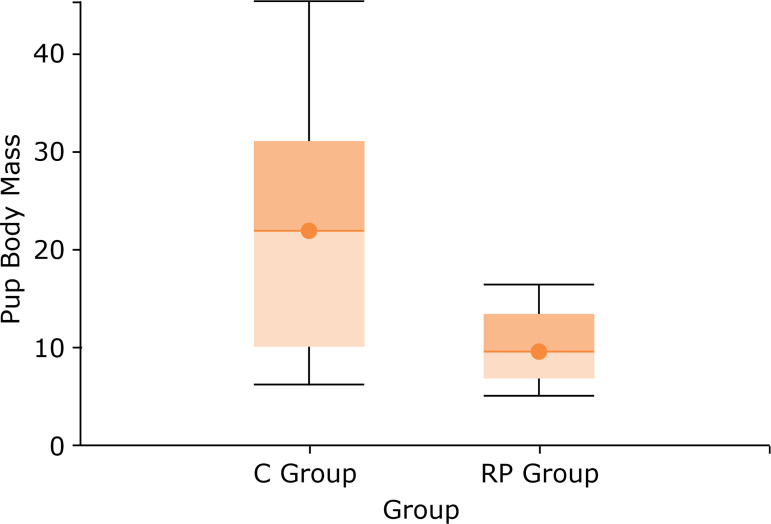
Box plot of pup body mass (in g). (p <0.01–Mann-Whitney test).

The pups’ body mass increase during lactation is shown in Fig. 1. At the end of this period, at 21 days of age, the body mass of RP group pups was only 39% of that of C group pups (p <0.01–Mann-Whitney test), as shown in Fig. 2.

C: control group; RP: reduced protein group.

C: control group; RP: reduced protein group.

**Figure 3:**
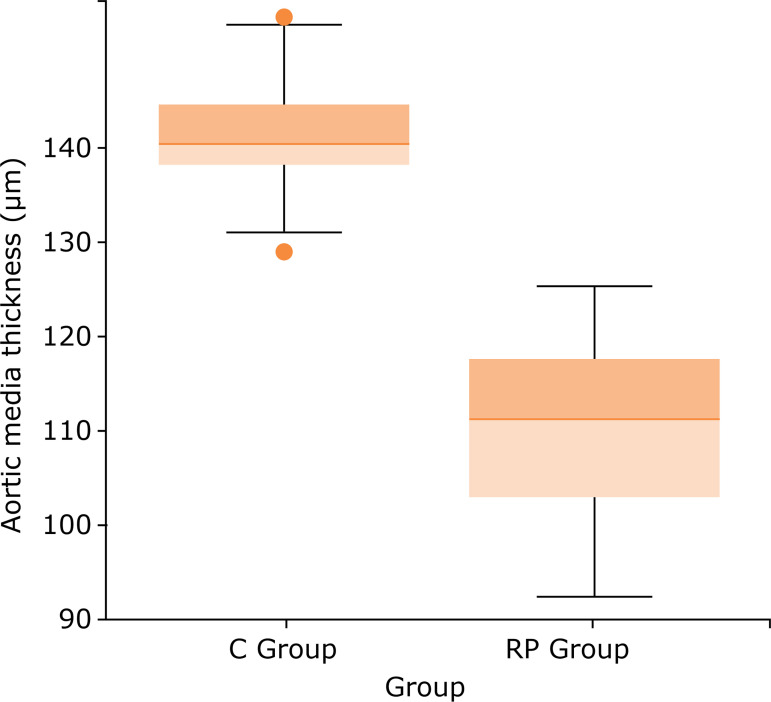
Box plot of pup aortic tunica media thickness in μm in the C and RP groups (p <0.01–Student’s t-test).

The descriptive analysis of the data of the thickness of the tunica media (μm) for the groups is presented in a very different way. Regarding the C group, we obtained mean of 141.27, median of 140.70, SD of 5.83, maximum value of 153.75 and minimum value of 129.10. In the RP group, we obtained mean of 110.70, median of 111.20, SD of 8.04, maximum value of 125.42 and minimum of 92.35. Figure 3 shows that the aortic tunica media thickness was significantly greater in C than in the RP group: 141.27 *vs*. 110.70 μm, respectively (p<0.01 – Student’s t-test).

C: control group; RP: reduced protein group.

**Figure 4:**
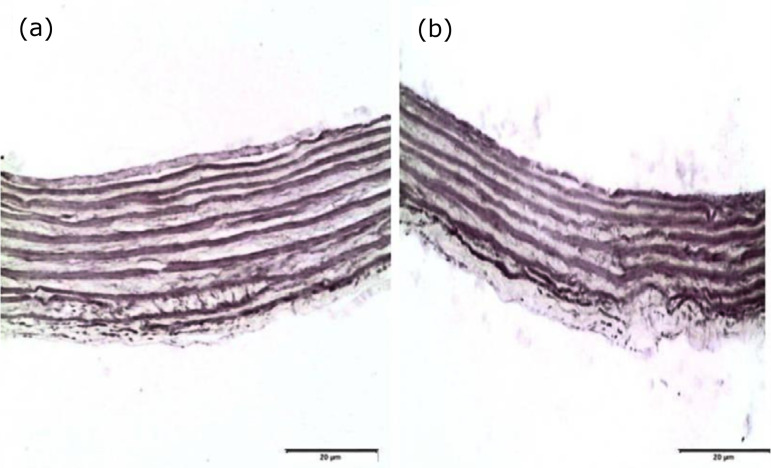
Photomicrograph of control group **(a)** and diet-restricted group **(b)** (x200 magnification).

Figure 4 histologically illustrates the quantitative results obtained. The difference in the thickness of the middle layer of the control group (a) and the protein-restricted group (b) is evident. No adverse events or death were observed.

Weigert’s Resorcin-Fuchsin stain.

## Discussion

Normal aortic development can be compromised by genetic factors, extreme metabolic fluctuation, or inadequate nutrition in fetal and neonatal life, including structural modification and possible functional failure^20^^,^ 21. During the literature review, we repeatedly found reports that aortic development begins during the prenatal phase and continues into the neonatal period. However, deficiency or supplementation of macro and micronutrients is often overlooked during lactation. This feeding period directly impacts the offspring’s constitution. Thus, the aim of this study was to analyze the effects of maternal protein malnutrition in Wistar rats on the offspring’s aortas. This animal is used as a model for studying the circulatory system, as well as for diseases of this system in humans. We used Weigert’s stain to perform a quantitative analysis of the thickness of the tunica media of the aorta using histomorphometric data. Under the guidance of CEUA-UNIFESP, we followed the principle of the 3Rs and employed one female per group, which was enough to demonstrate the effect of malnutrition on the offspring’s body mass and aorta.

Our results showed that, during lactation, RP group offspring did not gain weight, and the weight difference with controls was very expressive after 21 days. Our data corroborate several studies^22^^-^ 25 on maternal malnutrition during pregnancy and/or lactation in rats, which found significantly lower weight in the pups of mothers on restrictive diets after 21 days of lactation.

According to Hill *et al*.^20^, a low-iron diet in chicks up to 3 weeks of age had no effect on elastin or collagen content in relation to total proteins, despite the fact that the experimental group weighed less than controls. However, they found that fibrillin-3 was disorganized and less abundant, which could have caused the fragmentation of elastic fibers and elastic lamellae in diffuse networks. Besides the weight difference between the groups, the histological presentation of aortas in the RP group was distinct from controls. Aortic tunica media thickness was lower in the RP group, and there was lower deposition of extracellular matrix, suggesting that the mothers’ low-protein diet could have impacted postnatal collagen and elastin deposition.

In histopathological analysis, we found no numerical difference in elastic lamellae between the C and RP groups, which could be explained by the fact that embryonic development occurred under identical dietary conditions in both groups. Another rat study with similar conditions found no changes in the organization and distribution of elastic fibers in the trachea^26^. Thus, it could be that the postnatal development of the elastin and collagen deposition process is similar in both structures.

Animal studies on the influence of a low-protein diet during the prenatal and postnatal periods are needed to clarify the origin of this development and the ways neonates can be monitored to minimize the consequences in adulthood. A study involving a low-protein diet during the gestation and lactation of Wistar rats showed that the adult offspring of malnourished mothers have lower mitochondrial oxidative phosphorylation, lower antioxidant capacity and higher reactive oxygen species production. Thus, such a diet affects the development of heart disease in adulthood^27^.

The nutritional status with the highest risk of all-cause infant mortality is chronic malnutrition and low weight^28^. This risk is increased fivefold in children up to 12 months of age and eleven-fold in those over 12 months of age. Regardless of health care improvements, any environmental stress will affect infants whose clinical picture involves malnutrition interacting with infections^29^.

International strategies, especially in low- and middle-income countries, aim to reduce maternal, neonatal and child morbidity and mortality. One strategy are interventions during the first 1,000 days of life. According to English *et al*.^30^, the first 1,000 days of life encompass conception until the child’s second birthday, and this period has the greatest impact on the long-term health of both mother and child. One approach advocated during the prenatal period is micronutrient supplementation and balanced protein intake for weight maintenance in neonates and postpartum women. Kramer and Kakuma found that dietary protein restriction in mothers who are overweight or have high weight gain significantly reduced maternal weight gain as well as average birth weight^31^. The effects of maternal malnutrition could result in impaired child development and growth, a condition closely linked to the risk of developing hypertension and cardiovascular disease^32^.

Within the circulatory system, the aorta, the object of this study, is the largest and most important artery. Due to its elastic recoil, it can return to anatomical form after extension. Its elasticity is derived from elastic fibers, the main component of the extracellular matrix^33^. Elastin is a protein that forms an amorphous nucleus with surrounding microfibrillar glycoproteins, thus forming elastic fibers^34^^,^ 35. Microfibrils are formed from the polymerization of extracellular matrix proteins, especially fibrillin-1 and -2^36^. These proteins not only provide the structure necessary for elastic fibers, but also facilitate interaction with binding proteins^37^.

Elastin synthesis begins in the aorta during mid-gestation, when blood pressure rises^38^^,^ 39. The number of lamellae is defined during intrauterine development, and uniform growth and increased wall thickness, diameter and arterial length occur in postnatal development^40^.

Studies on changes in the aortic tunica media usually involve the ascending aorta. Few studies have considered the descending thoracic or abdominal aorta, since each segment has specific characteristics. Studies^39^^,^ 41 ^-^ 43 have shown that changes in the aortic tunica media are related to changes in transmural organization, which compromises the biomechanical properties of the aortic wall.

In clinical practice, such changes may lead to greater susceptibility in the microstructure of elastic lamellae, resulting in aortic wall degeneration and possible aortic aneurysm. Moreover, when medial elastic fibers are lost, they appear to be replaced with fibrous tissue, with smaller, more rigid areas that could favor a delamination process with consequent aortic dissection. Recent research, using imaging methods, correlated malnutrition in childhood with dilation of the thoracic aorta in adults in a Chinese population^44^.

## Conclusion

Our study found that modifying the diet of lactating mothers only in the postnatal period is sufficient to alter not only the body mass, but the development of vital structures in their offspring. Further research should verify these effects and analyze the involved structural and molecular components, determining which components are influenced by a maternal low-protein diet.
